# Leaving patients to their own devices? Smart technology, safety and therapeutic relationships

**DOI:** 10.1186/s12910-018-0255-8

**Published:** 2018-03-06

**Authors:** Anita Ho, Oliver Quick

**Affiliations:** 1 Centre for Applied Ethics, University of British Columbia, 227 – 6356 Agricultural Road, Vancouver, BC V6T 1Z2 Canada; 2 Bioethics Program, University of California, San Francisco, San Francisco, USA; 3 Ethics Services, Providence Health Care, Vancouver, Canada; 4 University of Bristol Law School, Wills Memorial Building, Bristol, BS8 1RJ UK

**Keywords:** Technology, Telemedicine, Patient safety, Medical ethics, Medical education, Professional regulation, Patient engagement

## Abstract

**Background:**

This debate article explores how smart technologies may create a double-edged sword for patient safety and effective therapeutic relationships. Increasing utilization of health monitoring devices by patients will likely become an important aspect of self-care and preventive medicine. It may also help to enhance accurate symptom reports, diagnoses, and prompt referral to specialist care where appropriate. However, the development, marketing, and use of such technology raise significant ethical implications for therapeutic relationships and patient safety.

**Main text:**

Drawing on lessons learned from other direct-to-consumer health products such as genetic testing, this article explores how smart technology can also pose regulatory challenges and encourage overutilization of healthcare services. In order for smart technology to promote safer care and effective therapeutic encounters, the technology and its utilization must be safe.

**Conclusion:**

This article argues for unified regulatory guidelines and better education for both healthcare providers and patients regarding the benefits and risks of these devices.

## Background

The use of technology in diagnosis and treatment is essential to safe and effective health care, although it may itself cause iatrogenic harm if not properly designed or used [1]. This paper explores the ethical implications of a specific type of information and communication technology on healthcare delivery: direct-to-consumer (DTC) self-monitoring devices and smartphone apps. Smart technology is now central to the vision of various healthcare systems towards more personalised care delivery [2], and may be especially useful in the context of an ageing population, an increasing prevalence of chronic conditions, and the goal of keeping patients out of hospitals. However, smart technologies may create a double-edged sword for therapeutic relationships and patient safety. On the one hand, when used properly, these technologies may promote safe and effective care delivery by empowering patients to take charge of their own health and promote efficient sharing of pertinent health information. On the other hand, if not regulated or incorporated appropriately into clinical care, smart technologies can pose significant ethical and safety concerns.

## Patient safety and smart technologies

Patient safety research is now firmly established on professional and political agendas across the world [3–6]. International studies estimate that between 8 to 12% of hospital admissions are associated with an adverse event, i.e., an unintended injury caused by medical management, rather than by the disease process [7]. As Merry and Brookbanks explain, an error occurs when someone tries to do the right thing but ends up doing the wrong thing [8]. Medication errors such as prescription of the wrong drug or dosage and missed or delayed diagnoses that result in a delay in referral and treatment are common problems [7]. In recent years, the study of adverse events and medical errors has evolved into a broader concern with safety -- a more expansive and somewhat nebulous term [9] which presents considerable challenges in terms of measurement [10] and regulation [11].

Adverse events and medical errors pose specific professional and bioethical implications regarding disclosure to patients as well as general ethical concerns around how modern healthcare is delivered and coordinated [12]. Advancing health technologies promise more cutting-edge diagnostic and treatment tools. Nonetheless, they also introduce new care and process complexities for multidisciplinary professionals. Given the basic bioethical tenets of beneficence and non-maleficence, finding reliable ways to track and prevent missed diagnoses is an important frontier for patient safety and health service research [13]. Connected to this are systematic failures around the communication of test results: some countries such as the UK rely on patients to chase up test results and to alert staff of missing and delayed results [14].

Various educational and communication strategies have been identified for improving diagnostic and prescription accuracy as well as reporting errors [15]. Information and e-communication technologies for professionals, such as electronic health records and computerized physician order entry, are already widely used [16]. Nonetheless, as monitoring and diagnostic technologies evolve in the age of person-centred care, can the use of smart technology by patients enhance both therapeutic relationships and patient safety? How can health systems and regulators ensure the appropriate marketing and use of such technology so that these devices can promote safe and efficient care organisation and delivery?

The use of technology by patients managing their health is not new. Patients with chronic conditions such as hypertension have long been able to purchase blood pressure monitors, and those with diabetes routinely carry out blood glucose tests. Other DTC tests can diagnose sexually transmitted infections [17, 18], detect pregnancy complications such as Group B strep [19], predict bowel cancer [20] and provide preliminary risk assessments for genetic conditions. Diagnostic and treatment algorithms, such as devices that can provide ECG reports via phone adaptors, and computer algorithms that can analyse photos of skin rashes and send suggested diagnosis and treatment options to patients via e-mail or SMS [21], are also increasingly available.

As smartphone and its associated technology continue to advance, device companies are increasingly targeting asymptomatic and pre-symptomatic populations, reaching not only patients but also healthy consumers. Half of the world’s adult population own a smartphone, and this is predicted to increase to 80% by 2020 [22]. With a low barrier to market entry, there are estimated to be over 100,000 mobile healthcare apps [23], including various DTC wellness wearables that continuously track vital signs or provide basic dietary and exercise activity information to users.

Smart technology has the potential for promoting ethical and effective care delivery in at least two ways. First, beneficence, or the promotion of patient well-being, is generally accepted as an important bioethical principle. Traditionally, symptomatic patients have to take several steps and rely exclusively on their physicians for direct information regarding their health. Many pre-symptomatic individuals do not know of their own susceptibility to various conditions and would have to wait until they have fallen ill before receiving medical attention or advice. Further delay of information ensues if laboratory tests are ordered. Patients in the UK often have to take the initiative to obtain their results [14], whereas many American physicians adopt the “no news is good news” approach and wait until they have received concerning laboratory reports before making appointments with their patients to discuss the findings [24]. Patients in both systems who do not hear back often wonder with anxiety whether their results are “normal” or whether their physicians simply have not received the reports. Worse yet, sometimes reports are missed and patients who require follow-up investigations or treatments are not notified [24, 25].

As health-tracking devices allow patients immediate access to their health data, they may enhance efficient and timely sharing of vital information to wider patient populations as well as facilitate more informed clinical counselling. Smart devices may help health professionals and patients in rural and isolated areas to share and coordinate recorded information that is traditionally unavailable to such communities, thereby promoting more equitable access to health information and corresponding management options. With smart devices, patients can ask more relevant and timely questions based on the recorded information, and physicians can confirm the accuracy of patients’ reports of their symptoms accordingly. Patients with busy lives or cognitive decline can particularly benefit, as they may recall their symptoms, activities, and other information incorrectly [26], especially when there is a significant time lapse between noticing the symptoms and consulting a physician [27]. At other times, patients’ physiological responses (e.g. heart rate) may fluctuate depending on various circumstances (e.g. work stress), such that their physiological markers at the time of their clinical consultation may not tell the full story. Having recorded information that spans a period of time may provide clinicians a more complete picture of the patient’s condition that can help to facilitate appropriate care and promote better health outcomes.

Second, the democratization of health information may allow consumers to access information for disease and illness prevention. More importantly, the direct availability of health information to patients has the potential of facilitating a more mutual therapeutic relationship, where informed patients can be actively involved in their care and treatment decisions [28]. By allowing patients and consumers to bypass traditional routes for accessing certain health information, DTC smart technology can potentially empower consumers in their everyday lives and patients in the care delivery process respectively. In the realm of clinical care, the rhetoric of patient- and family-centred care abound. Nonetheless, the notions of expertise and legitimacy continue to affect professionals’ willingness to take patients’ and families’ concerns seriously. As Coulter explains, patient expertise is often dismissed as a ‘fluffy notion that lacks the solid underpinning of scientific rigour on which medical care is supposedly built’ [29]. The tendency to under-value concerns expressed by patients and their caregivers can be regarded as a form of testimonial injustice [30], where too much or too little credibility is given to doctors’ or patients’ words because of prejudices about their respective roles. The utilization of smart monitoring technologies, especially if coupled with target counselling and education by healthcare professionals [31], may increase health literacy, empower patients to take a more active and informed role in the management of their own health, and bestow on patients more testimonial credibility. Connecting to our aforementioned argument from beneficence, if patients can present relevant and reliable data to validate their symptoms, they may be able to obtain speedier and more accurate diagnoses, thereby enhancing patient safety and well-being.

## Patient safety and ethical concerns regarding self-monitoring devices

While the increasing use of self-monitoring devices may facilitate patient engagement in the care delivery process, their value in promoting better health outcomes will depend on at least three significant factors.

First, the integrity and clinical utility of information from some DTC smart devices are currently questionable. As we learn from criticisms of DTC genetic tests, where individuals can send in cheek swabs to obtain genetic information regarding their risk of developing various conditions such as heart disease, diabetes, cancer, or Alzheimer’s, these test results may not be clinically meaningful or can be misinterpreted [32]. Medical devices are regulated by agencies such as the Food and Drug Administration (FDA) in the USA [33] and the Medicines and Healthcare Products Regulatory Agency (MHRA) in the UK [34]. Products which claim to diagnose, treat or prevent disease (including software applications) are likely to be classified as devices and regulated accordingly. Nonetheless, despite being aggressively marketed as health-promoting tools, wellness wearables which monitor general fitness are not formally categorised as medical devices and are thus not regulated. Limited studies exist regarding the accuracy or validity of various “symptom checker” apps, which are mostly developed by lay entrepreneurs rather than healthcare professionals [23]. There is currently no process of peer review beyond a simple anonymous user rating scale [35], even though these products may still present psychological and even physical risks to consumers and patients if they do not work as intended [36]. Such safety concerns raise questions of how regulatory and professional bodies should promote non-maleficence and beneficence as per these devices. Since some companies appeal to algorithmic authority to promote their apps to potential users [37] or purport to aid patients to self-diagnose with minimal evidence base, the potential for misuse is concerning [38]. Care providers may also have trouble keeping up with these developments, making it challenging for them to educate or warn patients accordingly. For example, the UK National Health Service (NHS) launched a pilot health apps library in 2013 and currently lists fourty-three apps as safe and trusted for patient use, raising questions about the safety and utility of other DTC devices [39].

Second, while meaningful data that can confirm one’s healthy status can reassure most patients, for the “worried well,” the device data may ironically exacerbate their health anxiety and compromise patient safety rather than promote productive engagement [40]. In the case of genetic tests, information about having or not having a particular gene mutation does not tell the whole story about one’s susceptibility to various conditions. The same applies to some biomarkers that need to be interpreted within the context of other variables. For example, a heart tracing that looks unusual for an asymptomatic person can be meaningless. However, in the absence of comprehensive patient and consumer education, healthy but anxious individuals may flock to their physicians and seek additional testing upon ambiguous results [41]. This can compromise therapeutic relationships, patient safety, and appropriate allocation of scarce medical resources. From over-prescription of proton-pump inhibitors to near-universal use of hormone-replacement therapy for postmenopausal women [42, 43], iatrogenic health risks from over-diagnosis and overtreatment abound [44]. The tide against medical paternalism may discourage clinicians from overtly dissuading consumers and patients from using health-tracking devices. Nonetheless, in the name of upholding patient autonomy, physicians may ironically feel pressured to practice “defensive medicine” and order further tests or low-value treatments that may carry other risks in order to avoid possible litigation [45]. Transferring the costs of follow-up testing and care from for-profit device companies to healthcare systems that are already overstretched also incur wasteful spending and pose justice considerations.

Third, by marketing directly to consumers, device companies are shifting the delicate but important balance in provider-patient relationships. If device users are primarily consumers rather than patients, a “buyer-beware” attitude may result, diminishing the importance of the therapeutic relationship. As Entwistle [46] argues, efforts to ‘activate’ individual patients to use various devices in the name of self-care “can be problematic if insufficient account is taken of patients’ own agendas, learning skills, and material and social circumstances.” An increasing expectation of patients to be actively and technologically engaged in their own care and an over-reliance on these applications regardless of patients’ desire, technological literacy, and economic means may violate patients’ autonomy and exacerbate access disparity [47]. It may marginalize patients who have no affordable or reliable access to internet or mobile technologies, such as people who live in rural or isolated areas, and affect others who feel uncomfortable with these technologies (e.g., elderly patients). Moreover, reliance on information from monitoring devices may ironically promote other forms of distrust. On the one hand, smart devices may render patients’ own testimony as being less credible, if their report cannot be supported by corresponding “objective” data. These concerns are evident in the treatment of chronic pain, where the pathology (e.g., lower back injury) does not always correspond to the reported severity by the patient [48]. Some physicians may thus question the truthfulness of the patient’s testimony regarding their symptoms, thereby reducing pain management ‘problems’ to doubts about patients’ trustworthiness [49]. On the other hand, patients who receive professional diagnoses that differ from what the devices suggest -- especially for the aforementioned worried well -- may distrust their physicians and their therapeutic recommendations. Under-treatment due to distrust of patients’ testimony and dismissal of professional advice based on patients’ over-reliance on these devices can both compromise therapeutic relationships and patient safety.

## Conclusion

Smart technology has the potential to facilitate patient engagement and strengthen the credibility of patient testimony, which may in turn help to promote patient safety and ethical therapeutic relationships. Nonetheless, the reliability and clinical utility of many of these devices have yet to be proven. There are also privacy and data confidentiality considerations that are beyond the scope of this brief debate article. To maximize the potential of these technologies, regulatory bodies need to develop clear and unified guidelines in distinguishing “recreational” from “medical” devices and monitor their marketing claims and safety respectively. For example, the utilization of a comprehensive risk assessment framework can help regulatory agencies to evaluate the complexity of an application and its probability and severity of harm [50]. Some agencies, such as the aforementioned Food and Drug Administration in the US [33] and the UK Medicines and Healthcare Products Regulatory Agency [34], are developing guidance on regulating and monitoring the efficacy of mobile medical applications. Creating a global infrastructure for mobile medical applications to provide common guidelines and serve as a repository for shared resources may also help to standardize safety requirements and promote evidence-based practices for patients [32].

Whilst the safety of medical devices is monitored by regulators, much of smart technology (e.g., wellness wearables) would fall outside of formal regulation, either because it is not classified as a medical device or because of enforcement discretion. Promotion of safe and appropriate use of these devices requires a more collaborative approach among different stakeholders. In particular, it requires the active educational and supervisory involvement of professional organisations and consumer/patient user experience feedback. In promoting non-maleficence and beneficence in the realm of smart technologies, professional organisations and patient advocacy groups can partner together to ensure that healthcare providers and consumers/patients are educated about the appropriate use and limits of these devices. Such collaboration may offer a more effective way to gather feedback and promote patient safety than formal regulations. For example, accredited continuing medical education training, which is widely available for various clinical devices, can be a model for educating clinicians regarding various smart technologies. It is only when these tools are marketed and utilised properly in the context of informed and supportive therapeutic relationships that they can effectively promote not only patient engagement, but also patient safety.

## Abbreviations

DTC: Direct-to-consumer
ECG: Electrocardiogram
FDA: Food and Drug Administration
MHRA: Medicines and Healthcare Products Regulatory Agency
NHS: National Health Service
SMS: Short message service
